# eVisits to primary care and subsequent health care contacts: a register-based study

**DOI:** 10.1186/s12875-024-02541-y

**Published:** 2024-08-12

**Authors:** Hanna Glock, Ulf Jakobsson, Beata Borgström Bolmsjö, Veronica Milos Nymberg, Moa Wolff, Susanna Calling

**Affiliations:** 1 Center for Primary Health Care Research, Department of Clinical Sciences Malmö, Lund University, Box 50332, Malmö, 202 13 Sweden; 2 University Clinic Primary Care Skåne, Region Skåne, Box 50332, Malmö, 202 13 Sweden

**Keywords:** e-visit, Virtual visit, Remote consultation, Telemedicine, Digital health, General practice, Primary health care, Access to primary care, Health care use

## Abstract

**Background:**

Evidence concerning health care use related to virtual visits is conflicting. More research has been called for regarding the effectiveness of text-based virtual visits (eVisits). Therefore, we investigated patient characteristics, diagnoses, and subsequent health care contacts after eVisits to primary care.

**Methods:**

We conducted a register-based cohort study of eVisits to an all-virtual public primary care unit in Sweden and subsequent health care contacts within 14 days. Data for 2021 were acquired from the regional health care databases. Diagnoses were sorted into relevant diagnostic groups, such as skin diagnoses and respiratory tract diagnoses. Multiple logistic regression was performed with subsequent health care contact as the outcome variable and diagnostic group for the eVisit as the predictor variable. Analyses were adjusted for age, sex, and socioeconomic index.

**Results:**

There were 5817 eVisits to a nurse and 4267 eVisits to a general practitioner (*N* = 10 084). Most patients were 20 to 39 years of age (41.8%). Skin diagnoses were most frequent (47.3%), followed by respiratory tract diagnoses (19.9%). Approximately one-fourth (25.8%) of the patients who completed an eVisit with a nurse or a general practitioner had a subsequent face-to-face visit within 14 days, mostly in primary care. Subsequent contacts were more frequent after an eVisit to a nurse than to a general practitioner. After an eVisit to a general practitioner, patients with infections (especially respiratory tract but also urinary tract) and unspecified diagnoses (especially skin-related) were more likely to require further health care contact compared to a group with various other diagnoses.

**Conclusions:**

eVisits to an all-virtual primary care unit may be appropriate for uncomplicated medical complaints. Nonetheless, the effectiveness of eVisits in terms of substitution of physical visits, and resource utilization in relation to the more complex care needs of a primary care population, should be further studied.

**Supplementary Information:**

The online version contains supplementary material available at 10.1186/s12875-024-02541-y.

## Background

After a gradual increase during the last decade, virtual visits to primary care were catalyzed by the COVID-19 pandemic [1–4]. The visits can be conducted via video, or via synchronous or asynchronous text [3, 5]. Expectations from policymakers and health care providers have been numerous and include increased access and improved efficiency [5, 6]. However, concerns have been raised regarding the effects on health care equity and resource use [5–7].

According to prior research, users of virtual visits to primary care are more commonly women, younger than 65 years of age and have high socioeconomic status [3, 8–10]. Recent systematic reviews indicate that clinical outcomes may be comparable to face-to-face visits, but more research is needed [8, 11, 12]. Regarding health care use related to virtual visits, the evidence is mixed [8, 11, 12]. Most prior studies have been conducted in the United States [2, 8, 11, 12]. Research has primarily concerned video visits, or covered several types of remote forms of contact, with fewer studies specifically on text-based visits.

A commonly used term for text-based visits is “eVisits”. In this paper, we define an eVisit as a synchronous or asynchronous two-way communication via a digital platform (app- or web-based). As of 2023, most Swedish primary care providers had introduced virtual visits, and more than half of the providers specifically offered or planned to offer eVisits via asynchronous chat [13, 14]. However, scientific publications regarding eVisits in a Swedish context were sparse.

More research has been called for regarding the effectiveness of eVisits, and to determine which conditions are suitable for eVisits [11]. One way to assess this may be through analysis of whether patients require additional health care contact following an eVisit. Prior studies vary in context and describe subsequent face-to-face contact rates from 5 to 25% after an eVisit to primary care [8, 11, 15–23]. Some register-based comparisons have been made with face-to-face visits, primarily regarding urinary tract infections and respiratory tract infections, finding similar subsequent health care contact frequencies [18–23]. However, there may be a selection bias when comparing virtual and face-to-face care, as the patients differ in characteristics and may also differ in seriousness of the condition [24]. No studies have investigated the need for follow-up depending on health care professional for the eVisit nor compared follow-up between different conditions when treated via eVisit. Such information could indicate seriousness of symptoms and if eVisits substitute physical visits, which would add to the ongoing discussion regarding the effectiveness of eVisits in different contexts.

The aim of this study was therefore to describe patient characteristics, diagnoses, and subsequent health care contact in a cohort of patients who completed an eVisit to a nurse or a physician in primary care in southern Sweden during 2021, and to analyze whether the need for subsequent health care contacts varied depending on diagnosis for the eVisit.

## Method

### Setting

Sweden has a regionalized health care system with universal health coverage. Swedish primary care is delivered at primary health care centers (PHCCs), which can be run through a regional public provider or through private providers who are publicly reimbursed. Skåne is the third largest region in Sweden by population, with 1.4 million inhabitants distributed between urban and rural areas in the southernmost part of the country [25].

In 2020, the public primary care provider in Region Skåne initiated an all-virtual unit for eVisits called Primary Health Care Skåne Online (PHC Online). The primary aim of the service was to provide eVisits to patients listed at a public PHCC in Region Skåne, as virtual visits were not otherwise provided by the PHCCs. However, the service was available to all inhabitants. The opening hours were weekdays 8am through 5pm and weekends 10am through 3pm. The service was operated separately from the physical PHCCs but was primarily staffed by general practitioners (GPs) and nurses who also worked at a public PHCC. Patients could be recommended to seek care at PHC Online when they got in contact with their public PHCC with a suitable complaint. They could also contact PHC Online directly without any prior health care contact. The service was only recommended for patients with certain types of complaints (allergies, skin conditions, urogenital problems, airways and infections, stomach and intestines, common childhood problems, and renewal of prescription). The visit was initiated with an automated digital anamnesis. Patients selected their type of complaint, and subsequent questions were prompted by the patients’ selection. Patients were also asked to fill out a general health profile. A nurse then initiated an eVisit through text communication that could be synchronous or asynchronous, primarily depending on whether the patient was available. If needed, the nurse offered the patient to continue the eVisit with a GP. In this paper, we refer to the eVisits as “with a nurse” or “with a GP” even though most eVisits with a GP were preceded by a contact with a nurse. Patients and staff could attach photos as part of the text-based communication. The nurse and the GP had the option to convert the visit into a video call, but the option was rarely used.

### Study design and participants

We conducted a register-based cohort study of the patients who completed an eVisit with PHC Online from February 19 to December 31, 2021. In addition to demographics and the Swedish version of the International Classification of Diseases (ICD-10-SE) diagnoses for the eVisit, we studied subsequent contacts with any health care facility in Region Skåne within 14 days after the eVisit. Furthermore, we analyzed whether subsequent health care contact rates differed depending on the diagnosis for the eVisit. We only included index eVisits, i.e., the patient’s first visit to PHC Online during the study period. Patients who had completed a face-to-face or virtual visit to a physician for the same diagnostic group within 14 days before the eVisit were excluded. All other index eVisits were analyzed, including eVisits where the patient may have been advised by the nurse or GP to seek physical care. The study was carried out in adherence with the Strengthening the Reporting of Observational Studies in Epidemiology (STROBE) guidelines [26].

### Data sources and variables

Pseudonymized datasets of eVisits to PHC Online, and health care contacts within 14 days before and after the eVisit, were acquired from Region Skåne’s Health Care Databases. Data did not include health care units outside of Region Skåne. Neither did data include diagnoses set by health care units that were not regionally funded (a minority of units, totaling 1% of Sweden’s health care costs [3]). Variables included age, sex, which PHCC the patient was registered at, type of health care contact, health care unit, health care professional and visit ICD-10 diagnoses.

Visit diagnoses were sorted into relevant diagnostic groups based on common complaints for patients using PHC Online: skin, urinary tract, respiratory tract, gastrointestinal, genital, eye, psychiatric, other, and unspecified diagnoses (ICD-10 codes that did not indicate symptom or diagnosis). As a measure of socioeconomic index, we acquired the Care Need Index (CNI) for the PHCC that the patient was registered at, i.e., as a clinic-level index [27]. The CNI includes seven different factors: single households > 65 years, children < 5 years, single parents, birthplace abroad, high mobility, low educational level, and unemployment. It is used in Sweden as a measure for the allocation of public primary care resources [27].

### Outcome variables

A subsequent health care contact was defined as any type of contact with a nurse or a physician at any health care level within 14 days after the index eVisit. Modes of subsequent health care contact included face-to-face visit, virtual visit, telephone contact, and other remote contact (virtual or letter). Health care levels included primary care, emergency unit, inpatient care, and specialist outpatient care. The subsequent contacts were grouped into outcome variables regarding different types of contact (mode of contact, health care level, health care professional, and whether the subsequent contact concerned the same diagnostic group as the eVisit) and dichotomized as “yes” or “no”, where “yes” indicated one or more subsequent contacts and “no” indicated no subsequent contact of the specified type.

### Sample size

The sample size was calculated to discern a 5 percentage point difference in subsequent health care contact rates between common diagnostic groups for the eVisit with an *α* level of 0.05 and 80% power. From preliminary data from PHC Online, we estimated the occurrence of common diagnostic groups (skin, urinary tract, and respiratory tract diagnoses) to be at least 10% of the study population for each diagnostic group. The follow-up rates in previous studies of 5–25% were used as an estimate [8, 11, 15–23]. Power calculations rendered a study size of approximately 4500 patients who had completed an eVisit with a nurse or a GP, respectively.

### Statistical methods

IBM SPSS Statistics (version 29) was used for statistical processing. Patient characteristics per diagnostic group, and characteristics of those who had subsequent health care contacts, were visualized through crosstabulations. Differences between groups were analyzed using Pearson’s chi-square test for categorical variables and one-way ANOVA test or Kruskal-Wallis test for continuous variables as appropriate. Post-hoc tests were conducted using the Bonferroni correction. Data were analyzed to ensure that assumptions for the use of logistic regression analysis were met.

Multiple logistic regression was performed with subsequent contact (yes/no) as the outcome variable and diagnostic group for the eVisit as the predictor variable. The diagnostic groups gastrointestinal, genital, eye, psychiatric, and other were merged into the group “all other diagnoses”. The group “all other diagnoses” was used as the reference as it constituted a varied selection of primary care diagnoses. As a subsequent analysis, the largest diagnostic groups (skin and respiratory tract diagnoses) were separated into subgroups. The regression models were adjusted for age, sex, and CNI of the patient’s registered PHCC. Type of health care professional for the eVisit was considered a mediator rather than a confounder of effects and was therefore not used for adjustment as the regression model was intended to study total effects (Additional file 1) [28]. Instead, analyses were conducted for all eVisits, and separately for eVisits to a nurse and to a GP. For goodness of fit, Nagelkerke R^2^ was reported and the Hosmer-Lemeshow test was applied.

Regarding the handling of missing data, this almost exclusively concerned diagnoses for index eVisits and subsequent health care contacts. Index eVisits that did not have a diagnosis were included in descriptive statistics but excluded from the analyses regarding follow-up as they lacked the predictor variable (diagnostic group). Considering the subsequent health care contacts, analysis regarding follow-up for the same diagnostic group as the index eVisit was only performed for the types of contacts where at least 95% had a diagnosis.

## Results

### Sample characteristics

**Fig. 1:**
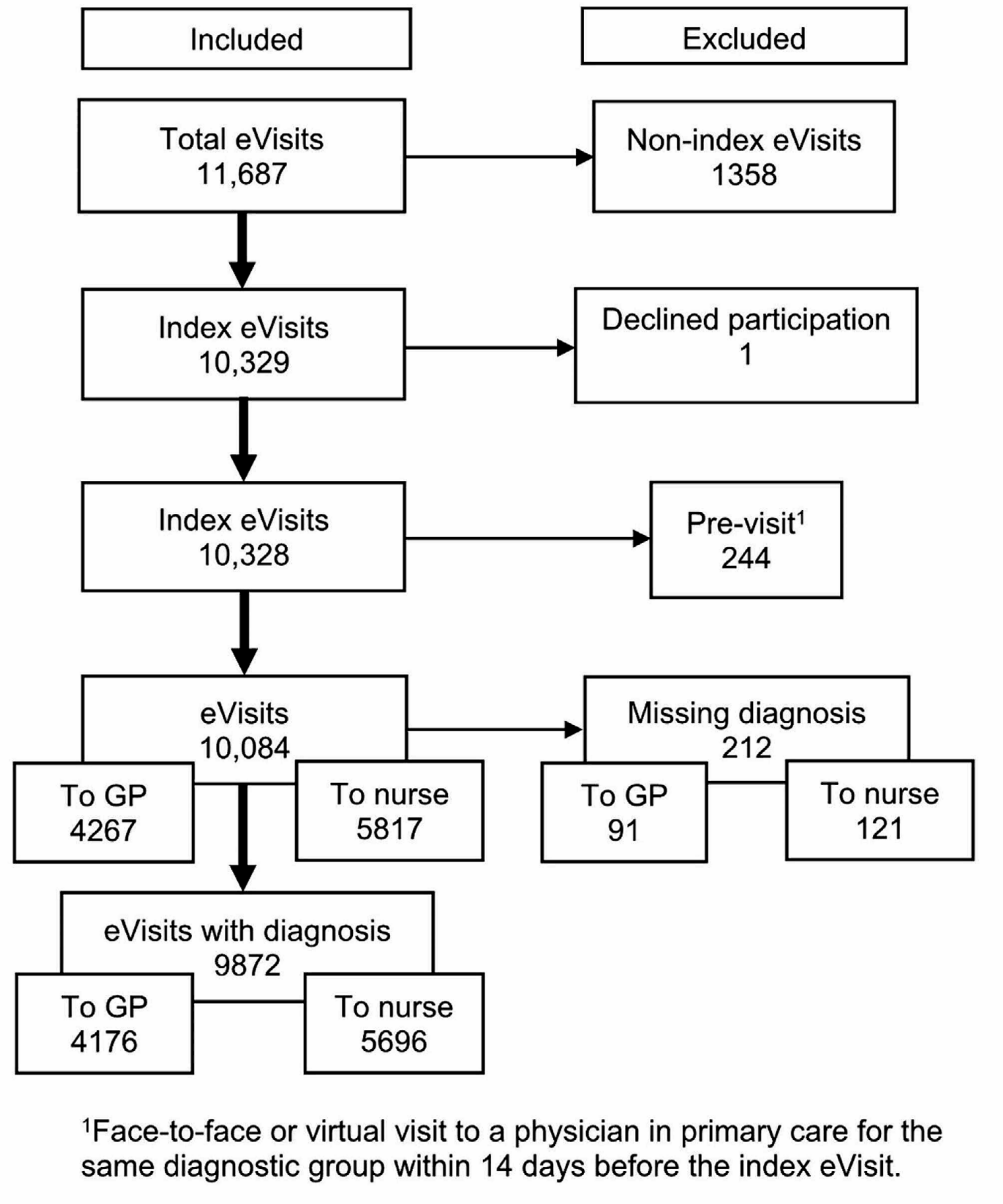
Flow chart of eVisits included and excluded at each stage of the study

Figure 1 illustrates the inclusion and exclusion of eVisits. A total of 10 084 eVisits were analyzed, of which 5817 were visits to a nurse and 4267 were visits to a GP (Fig. 1).

### eVisits

Patient characteristics are presented in Table 1. Women completed more eVisits than men. Considering age groups, young children aged 1 to 5 years (1051/10 084, 10.4%) and adults aged 20–39 years were the most frequent eVisitors, while few patients were 60 years of age or older. The median CNI for the patients’ registered PHCC was close to the median value for all PHCCs in the studied region. The most common diagnostic group was skin diagnoses, followed by respiratory tract diagnoses and urinary tract diagnoses. Less common diagnostic groups, such as gastrointestinal and eye diagnoses, were summarized into the group “all other diagnoses” (Table 1).

**Table 1:** Patient characteristics for index eVisits to a nurse, a general practitioner, and total

Characteristic	Index eVisit to nurse (*n* = 5817)	Index eVisit to GP (*n* = 4267)	Total index eVisits (*N* = 10 084)
Women, *n* (%)	3463 (59.5)	2730 (64.0)	6193 (61.4)
Age (years)			
Min-max	0–87	1–86	0–87
Median (Q1-Q3)	29 (18–42)	32 (20–46)	30 (19–44)
Age group, *n* (%)			
<20 years	1594 (27.4)	1049 (24.6)	2634 (26.1)
20–39 years	2539 (43.6)	1678 (39.3)	4217 (41.8)
40–59 years	1250 (21.5)	1173 (27.5)	2423 (24.0)
>59 years	434 (7.5)	367 (8.6)	801 (7.9)
CNI for PHCC^1^			
Min-max	1.27–7.13	1.27–7.13	1.27–7.13
Median (Q1-Q3)	2.40 (2.02–2.64)	2.39 (1.98–2.61)	2.40 (1.98–2.61)
Missing CNI, *n* (%)	205 (3.5)	136 (3.2)	341 (3.4)
Absolute CNI for PHCC^2^			
Median (Q1-Q3)	1.01 (0.85–1.11)	1.00 (0.83–1.10)	1.01 (0.83–1.10)
Diagnostic group, *n* (%)			
Skin	2890 (49.7)	1879 (44.0)	4769 (47.3)
Respiratory tract	1234 (21.2)	775 (18.2)	2009 (19.9)
Urinary tract	217 (3.7)	351 (8.2)	568 (5.6)
Unspecified^3^	810 (13.9)	431 (10.1)	1241 (12.3)
All other^4^	545 (9.4)	740 (17.3)	1285 (12.7)
No diagnosis	121 (2.1)	91 (2.1)	212 (2.1)

*Abbreviations* GP, general practitioner; CNI, care need index; PHCC, primary health care center

^1^CNI per person for 2021 for the patients’ registered PHCC

^2^(CNI for the patient’s registered PHCC)/(CNI for the median PHCC in Region Skåne)

^3^ICD-10 codes that did not indicate symptom or diagnosis, e.g., medical advice or prescription renewal

^4^All diagnoses except skin, respiratory tract, urinary tract, and unspecified diagnoses

Table 2 details patient characteristics per diagnostic group, including statistical and post hoc testing of differences between groups. Patients with urinary tract diagnoses were almost exclusively women and of older age compared to the other diagnostic groups. Patients with urinary tract diagnoses and “all other diagnoses” were more often handled by a GP (Table 2).

**Table 2:** Crosstabulations and statistical testing of different characteristics of index eVisits by diagnostic group (*n* = 9872)

Characteristic	Skin (*n* = 4769)	Respiratory tract (*n* = 2009)	Urinary tract (*n* = 568)	Unspecified^1^ (*n* = 1241)	All other^2^ (*n* = 1285)	*P* value
Women, *n* (%)	2745 (57.6)	1214 (60.4)	540 (95.1)	741 (59.7)	822 (64.0)	< .001^3,4^
Age in years, median (Q1-Q3)	29 (16–43)	31 (20–44)	38 (26–53)	32 (22–45)	30 (18–43)	< .001^5,6^
CNI for PHCC, median (Q1-Q3)^7^	2.39 (1.98–2.61)	2.39 (2.02–2.61)	2.42 (1.98–2.61)	2.40 (2.10–2.71)	2.40 (2.07–2.61)	.11^5^
Index eVisit to nurse, *n* (%)	2890 (60.6)	1234 (61.4)	217 (38.2)	810 (65.3)	545 (42.4)	< .001^3,8^

*Abbreviations* CNI, care need index; PHCC, primary health care center

^1^ICD-10 codes that did not indicate symptom or diagnosis, e.g., medical advice or prescription renewal

^2^All diagnoses except skin, respiratory, urinary, and unspecified

^3^Pearson’s chi-square test

^4^Significant at the 0.05 level with the Bonferroni correction: Urinary-skin, urinary-respiratory, urinary-unspecified, urinary-all other, all other-skin

^5^Kruskal-Wallis test

^6^Significant at the 0.05 level with the Bonferroni correction: Urinary-skin, urinary-respiratory, urinary-unspecified, urinary-all other, unspecified-skin, unspecified-respiratory, unspecified-all other

^7^CNI for the patients’ registered PHCC

^8^Significant at the 0.05 level with the Bonferroni correction: Urinary-skin, urinary-respiratory, urinary-unspecified, all other-skin, all other-unspecified, skin-unspecified

### Subsequent health care contact

All registered subsequent health care contacts are summarized in Additional file 2. Due to missing data on diagnosis for several types of contacts, only subsequent face-to-face visits to a physician could be analyzed regarding follow-up for the same diagnostic group as the eVisit.

Frequencies of different types of subsequent health care contacts are specified in Additional file 3. A total of 43.6% (4395/10 084) of the eVisits were followed by one or more remote or face-to-face health care contacts within 14 days. The frequency of subsequent face-to-face visits to a nurse or a physician was 25.8% (2606/10 084). Most of the subsequent contacts took place in primary care, with the most common in-person contact being a face-to-face visit to a primary care physician (1671/10 084, 16.6%). The frequency of emergency unit visits (160/10 084, 1.6%) and admissions to hospital (36/10 084, 0.4%) was low, especially if limited to the same diagnostic group as the eVisit (50/9872, 0.5% and 8/9872, 0.1%, respectively).

Characteristics of the eVisits that were followed by a subsequent health care contact are shown in Table 3. Women had a slightly higher frequency of subsequent contacts than men. Subsequent contacts were markedly more frequent after an eVisit to a nurse than to a GP, including subsequent face-to-face visits with 31.1% vs. 18.7%. Considering only eVisits to a GP, 11.4% of the patients had a subsequent face-to-face visit to a physician in primary care. If only subsequent visits for the same diagnostic group were considered, the follow-up frequency was 6.5% (Table 3).

**Table 3:** Characteristics of index eVisits by selected subsequent health care contacts within 14 days (*N* = 10 084)

Index eVisit characteristics^1^	*n*	Subsequent contact^2^	Subsequent remote contact^3^ with nurse or physician (*n* = 3523)	*P* value	Subsequent face-to-face visit to nurse or physician (*n* = 2606)	*P* value	Subsequent face-to-face visit to physician in primary care (*n* = 1671)	*P* value	Subsequent face-to-face visit to physician in primary care for same diagnostic group as eVisit *(n* = 1041)	*P* value
Health care professional for index eVisit, *n* (%)										
Nurse	5817	Yes	2293 (39.4)	< .001^4^	1809 (31.1)	< .001^4^	1184 (20.4)	< .001^4^	770 (13.2)	< .001^4^
GP	4267	Yes	1230 (28.8)		797 (18.7)		487 (11.4)		271 (6.4)	
Sex, *n* (%)										
Woman	6193	Yes	2272 (36.7)	< .001^4^	1690 (27.3)	< .001^4^	1055 (17.0)	.11^4^	645 (10.4)	.70^4^
Man	3891	Yes	1251 (32.2)		916 (23.5)		616 (15.8)		396 (10.2)	
Age in years, median (Q1-Q3)										
	NA	Yes	31 (20–46)	< .001^5^	31 (20–46)	< .001^5^	32 (20–47)	< .001^5^	32 (20–48)	.002^5^
	NA	No	29 (18–43)		30 (18–44)		30 (18–44)		30 (19–44)	
CNI for PHCC^6^, median (Q1-Q3)										
	NA	Yes	2.39 (2.00-2.61)	.02^5^	2.40 (2.04–2.64)	.10^5^	2.40 (2.06–2.64)	.32^5^	2.38 (1.98–2.61)	.28^5^
	NA	No	2.40 (2.07–2.61)		2.40 (2.02–2.61)		2.40 (2.02–2.61)		2.40 (2.03–2.61)	
Diagnostic group for index eVisit, *n* (%)										
Skin	4769	Yes	1418 (29.7)^9^	< .001^4^	1172 (24.6)^9^	< .001^4^	676 (14.2)^9^	< .001^4^	537 (11.3)^9^	< .001^4^
Respiratory tract	2009	Yes	803 (40.0)^10^		538 (26.8)^9,10^		405 (20.2)^10^		306 (15.2)^10^	
Urinary tract	568	Yes	249 (43.8)^10^		166 (29.2)^9,10^		115 (20.2)^10^		92 (16.2)^10^	
Unspecified^7^	1241	Yes	545 (43.9)^10^		377 (30.4)^10^		254 (20.5)^10^		5 (0.4)^11^	
All other^8^	1285	Yes	429 (33.4)^9^		302 (23.5)^9^		187 (14.6)^9^		101 (7.9)^12^	
No diagnosis	212	Yes	79 (37.3)^13^		51 (24.1)^13^		34 (16.0)^13^		0 (0.0)^13^	

*Abbreviations* GP, general practitioner; NA, not applicable; CNI, care need index, PHCC, primary health care center

^1^Percentages are row percentages, i.e., proportion of index eVisits with the specified characteristic which had a subsequent contact of the specified type

^2^Subsequent health care contact of the specified type within 14 days after the index eVisit

^3^Telephone, virtual text or video, or letter

^4^Pearson’s chi-square test

^5^Kruskal-Wallis test

^6^Care need index per person for 2021 for the patients’ registered primary health care center (PHCC).

^7^ICD-10 codes that did not indicate symptom or diagnosis

^8^All diagnoses except skin, respiratory tract, urinary tract, and unspecified

^9–12^Each number denotes a subset of diagnostic groups whose variable proportions do not differ significantly at 0.05 level with Bonferroni correction

^13^eVisits with no diagnosis were excluded from analysis of difference between groups

### Subsequent health care contact by diagnostic group

The number of subsequent health care contacts per diagnostic group are reported in Table 3, while the results of regression analyses are reported in Tables 4 and 5, and Additional file 4. The patients who had completed an eVisit for a respiratory tract diagnosis, a urinary tract diagnosis, or an unspecified diagnosis had an increased odds ratio for subsequent health care contacts compared to the group with “all other diagnoses” (Table 4). This was primarily evident after eVisits to a GP (Additional file 4 and Table 5). As shown in Table 5, the difference became more marked when the subsequent contact was specified to a visit to a physician in primary care for the same diagnostic group as the eVisit. Dividing the diagnostic groups into subgroups revealed markedly increased odds for subsequent health care contact after an eVisit to a GP for respiratory tract infections and unspecified skin diagnoses compared to the group “all other diagnoses” (Table 5). We did not divide urinary tract diagnoses into subgroups as the group almost exclusively consisted of urinary tract infections (539/568, 94.9%).

**Table 4:** Adjusted odds ratio^1^ for subsequent contact per diagnostic group (*n* = 9872)

		Subsequent health care contact within 14 days, AOR (95% CI)			
Diagnostic groups and subgroups for index eVisit	*n*	Remote contact^4^ with nurse or physician^5^	Face-to-face visit to nurse or physician^6^	Face-to-face visit to physician in primary care^7^	Face-to-face visit to physician in primary care for same diagnostic group as eVisit^8^
Skin^2^	4769	0.85 (0.75–0.98)	1.08 (0.93–1.25)	0.98 (0.82–1.16)	1.49 (1.19–1.86)
Skin infection^3^	840	0.78 (0.64–0.93)	0.90 (0.73–1.11)	0.89 (0.69–1.15)	1.40 (1.03–1.88)
Skin allergy or eczema^3^	728	0.49 (0.39–0.61)	0.60 (0.47–0.76)	0.48 (0.35–0.66)	0.67 (0.46–0.98)
Other skin^3^	855	0.66 (0.54–0.80)	0.83 (0.67–1.02)	0.66 (0.51–0.87)	0.92 (0.67–1.28)
Unspecified skin^3^	2346	1.11 (0.97–1.29)	1.44 (1.23–1.68)	1.31 (1.08–1.57)	2.02 (1.60–2.55)
Respiratory tract^2^	2009	1.33 (1.15–1.54)	1.20 (1.02–1.41)	1.48 (1.23–1.79)	2.10 (1.66–2.66)
Respiratory tract infection^3^	1458	1.56 (1.34–1.82)	1.51 (1.28–1.79)	1.97 (1.62–2.40)	2.87 (2.25–3.65)
Other respiratory^3^	551	0.85 (0.68–1.06)	0.53 (0.40–0.69)	0.43 (0.30–0.62)	0.40 (0.24–0.66)
Urinary tract^2^	568	1.41 (1.15–1.73)	1.21 (0.97–1.52)	1.40 (1.08–1.81)	2.16 (1.59–2.94)
Unspecified^2^	1241	1.54 (1.31–1.81)	1.40 (1.18–1.68)	1.48 (1.21–1.83)	0.05 (0.02–0.11)
All other^2,3^	1285	1.0 (Ref.)	1.0 (Ref.)	1.0 (Ref.)	1.0 (Ref.)

*Abbreviations* AOR; adjusted odds ratio

^1^Adjusted for age, sex, and care need index for the patient’s registered primary health care center

^2^Model with diagnostic groups

^3^Model with skin and respiratory tract diagnostic subgroups

^4^Telephone, virtual text or video, or letter

^5^Goodness of fit for model with diagnostic groups: Nagelkerke R^2^ = 0.026, Hosmer Lemeshow test (*X*^*2*^, *P* value) = 6.66 (0.57). For model with diagnostic subgroups: Nagelkerke R^2^ = 0.043, Hosmer Lemeshow test (*X*^*2*^, *P* value) = 10.15 (0.25)

^6^Goodness of fit for model with diagnostic groups: Nagelkerke R^2^ = 0.011, Hosmer Lemeshow test (*X*^*2*^, *P* value) = 3.91 (0.87). For model with diagnostic subgroups: Nagelkerke R^2^ = 0.034, Hosmer Lemeshow test (*X*^*2*^, *P* value) = 6.94 (0.54)

^7^Goodness of fit for model with diagnostic groups: Nagelkerke R^2^ = 0.014, Hosmer Lemeshow test (*X*^*2*^, *P* value) = 7.03 (0.53). For model with diagnostic subgroups: Nagelkerke R^2^ = 0.042, Hosmer Lemeshow test (*X*^*2*^, *P* value) = 10.18 (0.25)

^8^Goodness of fit for model with diagnostic groups: Nagelkerke R^2^ = 0.065, Hosmer Lemeshow test (*X*^*2*^, *P* value) = 21.30 (0.006). For model with diagnostic subgroups: Nagelkerke R^2^ = 0.10, Hosmer Lemeshow test (*X*^*2*^, *P* value) = 17.63 (0.024)

**Table 5:** Adjusted odds ratio^1^ for subsequent contact per diagnostic group after eVisit to a GP (*n* = 4176)

		Subsequent health care contact within 14 days, AOR (95% CI)			
Diagnostic groups and subgroups for index eVisit to GP	*n*	Remote contact^4^ with nurse or physician^5^	Face-to-face visit to nurse or physician^6^	Face-to-face visit to physician in primary care^7^	Face-to-face visit to physician in primary care for same diagnostic group as eVisit^8^
Skin^2^	1879	0.99 (0.81–1.20)	1.15 (0.91–1.45)	1.29 (0.95–1.76)	2.03 (1.33–3.10)
Skin infection^3^	516	0.95 (0.73–1.24)	1.14 (0.84–1.54)	1.31 (0.88–1.94)	2.20 (1.33–3.64)
Skin allergy or eczema^3^	580	0.69 (0.52–0.90)	0.88 (0.65–1.20)	0.94 (0.62–1.41)	1.36 (0.79–2.33)
Other skin^3^	451	0.86 (0.65–1.14)	0.95 (0.68–1.31)	0.95 (0.61–1.48)	1.31 (0.73–2.34)
Unspecified skin^3^	332	2.00 (1.52–2.65)	2.08 (1.52–2.85)	2.51 (1.70–3.71)	4.10 (2.49–6.76)
Respiratory tract^2^	775	1.49 (1.19–1.86)	1.41 (1.09–1.84)	1.92 (1.37–2.68)	2.82 (1.80–4.44)
Respiratory tract infection^3^	514	1.63 (1.27–2.08)	1.73 (1.30–2.29)	2.55 (1.80–3.61)	4.15 (2.62–6.57)
Other respiratory^3^	261	1.23 (0.90–1.70)	0.86 (0.57–1.30)	0.82 (0.47–1.44)	0.52 (0.20–1.37)
Urinary tract^2^	351	1.59 (1.20–2.11)	1.47 (1.06–2.04)	1.73 (1.15–2.61)	2.62 (1.54–4.47)
Unspecified^2^	431	1.95 (1.51–2.52)	1.67 (1.24–2.25)	2.43 (1.69–3.50)	0.12 (0.03–0.52)
All other^2,3^	740	1.0 (Ref.)	1.0 (Ref.)	1.0 (Ref.)	1.0 (Ref.)

*Abbreviations* GP, general practitioner; AOR, adjusted odds ratio

^1^Adjusted for age, sex, and care need index for the patient’s registered primary health care center

^2^Model with diagnostic groups

^3^Model with skin and respiratory tract diagnostic subgroups

^4^Telephone, virtual text or video, or letter

^5^Goodness of fit for model with diagnostic groups: Nagelkerke R^2^ = 0.028, Hosmer Lemeshow test (*X*^*2*^, *P* value) = 21.92 (0.005). For model with diagnostic subgroups: Nagelkerke R^2^ = 0.046, Hosmer Lemeshow test (*X*^*2*^, *P* value) = 2.66 (0.95)

^6^Goodness of fit for model with diagnostic groups: Nagelkerke R^2^ = 0.012, Hosmer Lemeshow test (*X*^*2*^, *P* value) = 4.63 (0.80). For model with diagnostic subgroups: Nagelkerke R^2^ = 0.027, Hosmer Lemeshow test (*X*^*2*^, *P* value) = 9.20 (0.33)

^7^Goodness of fit for model with diagnostic groups: Nagelkerke R^2^ = 0.018, Hosmer Lemeshow test (*X*^*2*^, *P* value) = 13.79 (0.09). For model with diagnostic subgroups: Nagelkerke R^2^ = 0.039, Hosmer Lemeshow test (*X*^*2*^, *P* value) = 9.60 (0.29)

^8^Goodness of fit for model with diagnostic groups: Nagelkerke R^2^ = 0.047, Hosmer Lemeshow test (*X*^*2*^, *P* value) = 4.74 (0.79). For model with diagnostic subgroups: Nagelkerke R^2^ = 0.083, Hosmer Lemeshow test (*X*^*2*^, *P* value) = 4.05 (0.85)

## Discussion

### Main findings

This study of patient characteristics, diagnoses, and subsequent health care contacts after eVisits to an all-virtual public primary care unit found that women and young to middle-aged individuals were the most frequent visitors, and that skin conditions were the most common diagnostic group. Approximately one-fourth (25.8%) of the patients who completed an eVisit with a nurse or a GP had a subsequent face-to-face visit within 14 days, mostly in primary care. Subsequent contacts were more frequent after an eVisit to a nurse than to a GP. After an eVisit to a GP, patients with infections (especially respiratory tract but also urinary tract) and unspecified diagnoses (especially skin-related) were more likely to require further health care contact compared to a group with various other diagnoses.

### Strengths and limitations

PHC Online was one of the first services for eVisits to public primary care, and it was introduced in Sweden’s third most populous region, which increases the novelty as well as the generalizability of the results. Another strength of this study was that we used a large dataset obtained from a regional claims database that is also maintained for research purposes. We studied all patients who completed a visit with PHC Online during almost one year, thus accounting for seasonal variations. Furthermore, we described several types of subsequent health care contacts, providing a comprehensive picture of health care use after eVisits. Finally, we analyzed subsequent contacts for the same diagnostic group as the eVisit, which increases precision.

Considering the demographics of the study population, comparably few participants were older than 60 years of age. Thus, our results are primarily valid for patients under 60 years of age, i.e., the age group that currently uses virtual care. Furthermore, patients were instructed to only use the service for certain complaints, which excluded patients with other types of complaints. It should also be noted that the study took place in 2021, during the latter stages of the COVID-19 pandemic. Thus, the selection of patients may have differed from non-pandemic conditions. These factors decrease generalizability.

Regarding missing data, a diagnosis was lacking in a significant proportion of all subsequent contacts with a nurse as well as in subsequent remote contacts with a physician. Consequently, for these types of subsequent contacts, we could not make any conclusions regarding follow-up for the same diagnostic group as the eVisit. The other main type of missing data was diagnosis for the eVisit. However, this concerned a limited number of patients (2.1%; 212/10084) with similar characteristics as the total study population and should not have affected the outcome.

The measure of socioeconomic standard, CNI, was acquired for the patient’s registered PHCC. Thus, it was a crude measure on a group level. It may suffice to partially control for socioeconomics in statistical analysis but should not be further interpreted as a descriptive statistic.

### Findings in relation to prior research

The patients seeking PHC Online had similar demographic characteristics as patients in other studies of virtual visits including eVisits, with a higher proportion of younger patient groups [3, 8–10]. This thus remains an issue of concern in terms of equal access to health care [9].

Skin diagnoses were even more common in our study population than in prior research, while respiratory tract and urinary tract diagnoses (primarily infections) were common in accordance with other studies [11, 15, 29]. These are often simple and potentially self-healing conditions, in line with qualitative research reporting that patients choose to use virtual visits for less severe conditions [30]. This may indicate a suboptimal use of health care resources [7].

Prior research on subsequent health care contacts after eVisits is limited. Most studies focus on urinary or respiratory tract infections. Studies differ regarding health care professional for the eVisit, time period, and type of subsequent contact. In these studies, subsequent face-to-face visit rates ranged from 5 to 25% [15–23]. In our study, subsequent face-to-face contacts were within the same range, from the most specific type of follow-up (with a physician in primary care after an eVisit to a GP for the same diagnostic group) at 6% to the follow-up frequency with nurse or physician for all patients for any reason at 26%. Our results thus show similar follow-up rates as prior research when considering a more varied array of diagnoses – and indicate the importance of the choice and detailing of outcome measure.

What our study primarily adds is the finding that the PHC Online patients had a higher frequency of subsequent health care contacts after an eVisit to a nurse than to a GP. Our analysis included all eVisits to PHC Online, regardless of whether the patient was advised by the nurse or the GP to seek physical care. Therefore, a reasonable explanation for our results could be that a larger proportion of the eVisits to a nurse resulted in the patient being recommended to seek a physical unit. Such visits should rather be considered triage. If so, more specific instructions to patients regarding which types of complaints are suitable for an eVisit may decrease avoidable triage contacts.

Prior studies indicate that face-to-face follow-up rates after virtual and face-to-face visits to primary care do not differ [11, 16, 19–23]. However, these were also registry-based studies, which means that the results must be interpreted with caution. Virtual care-seekers are generally healthier and thus should require less follow-up [3, 8–10, 30]. As an alternative analysis, we instead compared follow-up between eVisits for different diagnostic groups. We found that patients with infections (especially respiratory tract but also urinary tract) and unspecific complaints (especially skin-related) were more likely to require further health care contact after an eVisit with a GP than patients with various other diagnoses. This finding calls for cautious interpretation. It could indicate conditions that should be referred directly to physical care, due to seriousness of the condition or inappropriateness of the eVisit setting. However, it could also indicate actual needs that eVisits can help attend to where some follow-up is always expected, as opposed to supply-induced demand [7].

Finally, it should be noted that we did not study virtual visits with the patient’s own GP. Thus, results could be generalized to eVisits conducted within the same health care system for minor illnesses, but not to virtual visits as an integral part of a primary care with continuity. Relating to prior research, telephone or video visits with the patient’s own GP have been shown to decrease emergency department use compared to telephone or video visits with any GP [31].

### Further research

Areas for future register-based studies include continuity of care, where the outcomes of eVisits provided by the patient’s own GP, integrated with the patient’s PHCC, and separate from it could be compared. A pertinent next step in the research on eVisits would be studies using a randomized controlled trial design for comparison with physical visits and telephone consultations. Such a design would counter selection bias and increase the ability to draw conclusions about causation. Possible outcome measures would be follow-up contacts, quality measures such as prescription of antibiotics, patient experience, and health care expenditure. In a randomized controlled trial design, it would also be possible to compare outcomes between different symptoms or diagnoses. Such studies could inform policy decisions on how to use digital care to meet needs but not to induce demand.

## Conclusions

eVisits to an all-virtual public primary care unit primarily attracted younger patient groups, appeared to partially serve a triage function when handled by a nurse and often concern simple conditions also when managed by a GP. Most patients did not require follow-up, and those who did mainly remained in primary care. Our results thus indicate that this type of eVisits may be appropriate for uncomplicated medical complaints. Nonetheless, the effectiveness of eVisits in terms of substitution of physical visits, and resource utilization in relation to the more complex care needs of a primary care population, should be further studied.

### Electronic supplementary material

Below is the link to the electronic supplementary material.

**Additional file 1:** Causal diagram of the total effects of diagnostic group on follow-up adapted from DAGitty.net [28]

**Additional file 2:** Flow chart of registered subsequent health care contacts within 14 days after the index eVisit

**Additional file 3:** Types of subsequent health care contacts within 14 days after the index eVisit (N=10 084)

**Additional file 4:** Adjusted odds ratio^1^ for subsequent contact per diagnostic group after eVisit to a nurse (n = 5696)

## Data Availability

The datasets used and analyzed during the current study are available from the corresponding author upon reasonable request.

## Abbreviations

AOR: Adjusted Odds Ratio
CNI: Care Need Index
eVisits: Text-based virtual visits
GP: General Practitioner
ICD: International Classification of Diseases
PHCC: Primary Health Care Center
PHC Online: Primary Health Care Skåne Online
