# Supplementary material for: eVisits to primary care and subsequent health care contacts: a register-based study
Source: BMC Prim Care. 2024 Aug 12;25:297. doi: 10.1186/s12875-024-02541-y (PMC11318178; doi:10.1186/s12875-024-02541-y)
Supplement: Supplementary file 1 — Additional file 1: Causal diagram of the total effects of diagnostic group on follow-up adapted from DAGitty.net [28] [file 12875_2024_2541_MOESM1_ESM.docx]

SexSexAgeAgeCare need indexCare need indexHealth care professional for eVisitHealth care professional for eVisitDiagnostic group for eVisitDiagnostic group for eVisitSubsequent health care contact within 14 daysSubsequent health care contact within 14 days
