# Supplementary material for: eVisits to primary care and subsequent health care contacts: a register-based study
Source: BMC Prim Care. 2024 Aug 12;25:297. doi: 10.1186/s12875-024-02541-y (PMC11318178; doi:10.1186/s12875-024-02541-y)
Supplement: Supplementary file 2 — Additional file 2: Flow chart of registered subsequent health care contacts within 14 days after the index eVisit [file 12875_2024_2541_MOESM2_ESM.docx]

Total subsequent contacts

9642

No diagnosis:

3100 (32%)

Inpatient care

44

No diagnosis:

1 (2%)

Unique individuals

4394

Contacts per individual:

1-19

Subsequent contact with nurse

5221

No diagnosis:

2211 (42%)

Subsequent contact with physician

4421

No diagnosis:

889 (20%)

Digital visit

271

No diagnosis:

51 (19%)

Physical visit

1125

No diagnosis:

217 (19%)

Other remote

482

No diagnosis:

284 (59%)

Telephone

3328

No diagnosis:

1657 (50%)

Other remote

1052

No diagnosis:

620 (59%)

Telephone

594

No diagnosis:

148 (25%)

Digital visit

318

No diagnosis:

47 (15%)

Physical visit

2448

No diagnosis:

69 (3%)

Primary care

463

No diagnosis:

284 (61%)

Primary care

3239

No diagnosis:

1640 (51%)

Primary care

271

No diagnosis:

51 (19%)

Primary care

875

No diagnosis:

186 (21%)

Primary care

1030

No diagnosis:

609 (59%)

Primary care

521

No diagnosis:

143 (27%)

Primary care

1859

No diagnosis:

47 (3%)

Primary care

318

No diagnosis:

47 (15%)

Secondary care

384

No diagnosis:

15 (4%)

Emergency unit

199

No diagnosis:

7 (4%)
