# Supplementary figures and images for: eVisits to primary care and subsequent health care contacts: a register-based study
Source: BMC Prim Care. 2024 Aug 12;25:297. doi: 10.1186/s12875-024-02541-y (PMC11318178; doi:10.1186/s12875-024-02541-y)

**
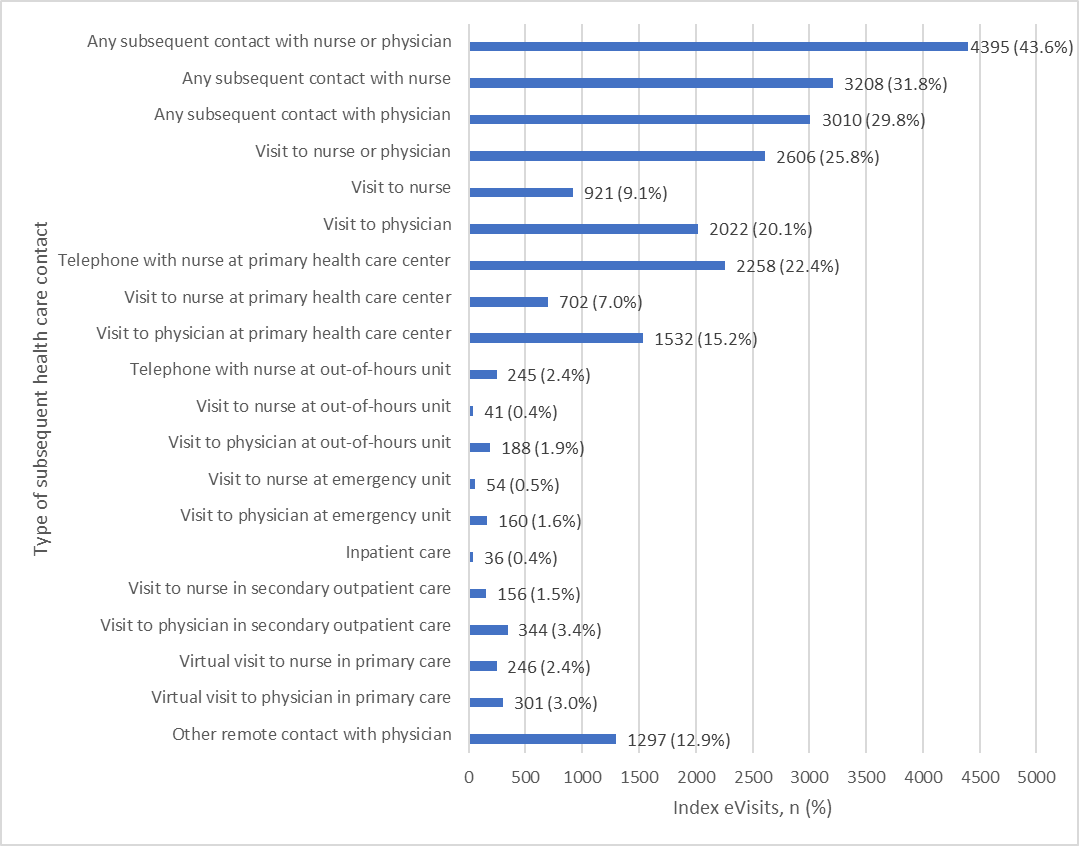
**

Supplement: Supplementary file 3 — Additional file 3: Types of subsequent health care contacts within 14 days after the index eVisit (N=10 084) [file 12875_2024_2541_MOESM3_ESM.docx]
