# Supplementary material for: eVisits to primary care and subsequent health care contacts: a register-based study
Source: BMC Prim Care. 2024 Aug 12;25:297. doi: 10.1186/s12875-024-02541-y (PMC11318178; doi:10.1186/s12875-024-02541-y)
Supplement: Supplementary file 4 — Additional file 4: Adjusted odds ratio1 for subsequent contact per diagnostic group after eVisit to a nurse (n = 5696) [file 12875_2024_2541_MOESM4_ESM.docx]

**Additional file 4** Adjusted odds ratio^1^ for subsequent contact per diagnostic group after eVisit to a nurse (*n* = 5696).

|  |  | **Subsequent health care contact within 14 days, AOR (95% CI)** | | | |
| --- | --- | --- | --- | --- | --- |
| **Diagnostic groups and subgroups for index eVisit to nurse** | ***n*** | **Remote contact^4^ with nurse or physician^5^** | **Face-to-face visit to nurse or physician^6^** | **Face-to-face visit to physician in primary care^7^** | **Face-to-face visit to physician in primary care for same diagnostic group as eVisit^8^** |
| Skin^2^ | 2890 | 0.61 (0.51-0.74) | 0.80 (0.66-0.97) | 0.66 (0.53-0.83) | 1.03 (0.79-1.34) |
| Skin infection^3^ | 324 | 0.64 (0.48-0.86) | 0.80 (0.59-1.09) | 0.75 (0.53-1.06) | 1.21 (0.82-1.80) |
| Skin allergy or eczema^3^ | 148 | 0.40 (0.27-0.60) | 0.52 (0.34-0.80) | 0.29 (0.16-0.54) | 0.52 (0.27-1.00) |
| Other skin^3^ | 404 | 0.47 (0.35-0.61) | 0.67 (0.50-0.89 | 0.48 (0.34-0.68) | 0.71 (0.47-1.07) |
| Unspecified skin^3^ | 2014 | 0.65 (0.54-0.79) | 0.85 (0.70-1.05) | 0.72 (0.57-0.90) | 1.10 (0.84-1.45) |
| Respiratory tract^2^ | 1234 | 0.97 (0.80-1.19) | 0.85 (0.68-1.05) | 1.02 (0.81-1.30) | 1.46 (1.10-1.94) |
| Respiratory tract infection^3^ | 944 | 1.15 (0.93-1.42) | 1.07 (0.86-1.34) | 1.34 (1.05-1.70) | 1.90 (1.42-2.53) |
| Other respiratory^3^ | 290 | 0.54 (0.40-0.73) | 0.31 (0.22-0.46) | 0.24 (0.15-0.40) | 0.30 (0.16-0.55) |
| Urinary tract^2^ | 217 | 1.36 (0.99-1.88) | 1.13 (0.81-1.56) | 1.37 (0.96-1.95) | 2.27 (1.54-3.36) |
| Unspecified^2^ | 810 | 1.03 (0.83-1.28) | 0.95 (0.76-1.20) | 0.90 (0.69-1.16) | 0.02 (0.01-0.07) |
| All other^2,3^ | 545 | Ref. | Ref. | Ref. | Ref. |

Abbreviations: GP, general practitioner; AOR, adjusted odds ratio.

^1^Adjusted for age, sex, and care need index for the patient’s registered primary health care center.

^2^Model with diagnostic groups.

^3^Model with skin and respiratory tract diagnostic subgroups.

^4^Telephone, virtual text or video, or letter.

^5^Goodness of fit for model with diagnostic groups: Nagelkerke R^2^ = 0.034, Hosmer Lemeshow test (*X^2^*, *P* value) = 7.26 (.51). For model with diagnostic subgroups: Nagelkerke R^2^ = 0.044, Hosmer Lemeshow test (*X^2^*, *P* value) = 7.31 (.51).

^6^Goodness of fit for model with diagnostic groups: Nagelkerke R^2^ = 0.018, Hosmer Lemeshow test (*X^2^*, *P* value) = 4.07 (.85). For model with diagnostic subgroups: Nagelkerke R^2^ = 0.034, Hosmer Lemeshow test (*X^2^*, *P* value) = 12.21 (.14).

^7^Goodness of fit for model with diagnostic groups: Nagelkerke R^2^ = 0.022, Hosmer Lemeshow test (*X^2^*, *P* value) = 4.76 (.78). For model with diagnostic subgroups: Nagelkerke R^2^ = 0.046, Hosmer Lemeshow test (*X^2^*, *P* value) = 6.49 (.59).

^8^Goodness of fit for model with diagnostic groups: Nagelkerke R^2^ = 0.088, Hosmer Lemeshow test (*X^2^*, *P* value) = 10.66 (.22). For model with diagnostic subgroups: Nagelkerke R^2^ = 0.111, Hosmer Lemeshow test (*X^2^*, *P* value) = 8.85 (.36).
